# Does Medicare Advantage funding disparity affect managed care insurers' profitability? Evidence from Puerto Rico

**DOI:** 10.1093/haschl/qxag242

**Published:** 2026-09-04

**Authors:** Sergio A Rivera-Rodríguez, Naomi Zewde, Alexandra C Rivera-González

**Affiliations:** Department of Health Policy and Management, CUNY Graduate School of Public Health & Health Policy, New York, NY 10027, United States; Department of Health Policy and Management, University of California, Los Angeles, CA, United States; Department of Public Health, University of California, Merced, CA, United States

**Keywords:** medicare advantage, Puerto Rico, health insurance, funding disparity, managed care

## Abstract

**Introduction:**

Puerto Rico's Medicare Advantage market operates under a persistent yet fluctuating federal funding disparity, with reported benchmark payments approximately 38% below those in US states. Health insurance industry representatives have argued that this disparity constrains their financial margins, warranting measures to reduce healthcare benefits and demand more federal funds for Medicare.

**Methods:**

This study assessed the relationship between Puerto Rico's Medicare funding disparity and medical loss ratio, administrative loss ratio, and profit margins within the Medicare Advantage market. We analyzed insurer financial statements filed with the Puerto Rico Insurance Commissioner Office and Part C plan payment data from the Centers for Medicare & Medicaid Services using descriptive trends and regression models.

**Results:**

The funding disparity was not significantly associated with medical loss ratio or profit margin, but was associated with higher administrative loss ratio. Profit margins exhibit abrupt fluctuations coinciding with external shocks affecting healthcare utilization and the healthcare market.

**Conclusion:**

Annual variation in the funding disparity alone does not explain insurer financial performance. Efforts to increase federal Medicare Advantage payments to Puerto Rico should be accompanied by oversight regarding how additional revenues are allocated among medical care, administrative activities, and profits.

## Introduction

Throughout the years, there has been a steady shift toward delivering Medicare using Medicare Advantage (MA), managed by private managed care insurers, rather than the Traditional Medicare program, managed by the federal government. In the United States, only 17% of Medicare beneficiaries were enrolled in MA in 2007, compared to 54% in 2024.^1^ For Puerto Rico (PR), a US territory, MA enrollment rates far exceed the national average, with 95% of Medicare beneficiaries enrolled in a MA plan in 2024 compared to 54% in the United States.^1^ Although the MA managed care model aims to reduce healthcare costs while preserving a high quality of care,^2^ some have questioned if MA plans are achieving these goals. Compared to Traditional Medicare, Medicare per capita spending is higher for MA programs, in large part due to the greater administrative costs.^2-8^ Meanwhile, as private entities, companies administering MA plans are able to generate profit.^9^ These trends provide an important context for understanding why closer study of the MA program in Puerto Rico is warranted.

Despite being a federal program, Medicare in Puerto Rico receives less funds than states due to the territorial status of the archipelago.^10^ Risk-standardized benchmark payments to MA plans in Puerto Rico are approximately 38% less than those in US states.^10^ However, 1 study found that higher risk scores and quality bonus payments partially offset this gap, reducing the difference in realized federal payments to roughly 26%.^10^ Importantly, provider payment rates in Puerto Rico are also lower, which can create room for insurers to maintain profitability even under conditions of relative underfunding. For example, Medicare reimbursement rates for physicians in Puerto Rico are 40% lower than in the US states.^11^ The impact of Medicare funding disparity on insurer financial performance in Puerto Rico has become the centerpiece of a debate.

The Medicaid and Medicare Advantage Product Association of Puerto Rico, an organization that consolidates the 6 health insurance companies that manage Medicaid and Medicare in Puerto Rico, have argued that federal underfunding of Medicare in the territory has contributed to unsustainable payment rates and financial instability in the health insurance sector.^12^ These claims have prompted consideration of measures to reduce healthcare benefits and demand more federal funds for MA beneficiaries.^12,13^ However, different economic analyses of Puerto Rico's health insurance sector have documented that insurers have maintained consistent profitability regardless of funding disparity and that, with highly concentrated ownership, they operate as an oligopoly sustained by public funds.^14-16^ This disagreement presents an empirical question: do year-to-year changes in Puerto Rico's Medicare funding gap move along insurer profit margins or do profits fluctuate independent of federal payment levels?^15^

This study analyzes whether year-to-year changes in Puerto Rico's Medicare funding are associated with fluctuations in medical loss ratio (MLR), administrative loss ratio (ALR), and profits in the MA market. We assess the extent to which profit margins appear to track federal payment disparities vs external shocks (eg, Hurricane María and the COVID-19 pandemic) and market-structure changes (eg, Affordable Care Act [ACA] and MA acquisitions) against 4 sets of expectations. First, if funding discrepancies between Puerto Rico and the United States influence profit margins, we would expect wider funding gaps to be aligned with lower profits and narrower gaps in funding with higher profits in Puerto Rico's MA market. Second, in 2014, the ACA introduced MA benchmark reductions and an 85% MLR floor, which we expect to appear as a structural break that can impact profit margins.^17^ Third, Hurricane María (2017) and the COVID-19 pandemic (2020) represent utilization shocks; there was a lower utilization rate of healthcare services while insurers continued receiving capitated federal payments in Medicare.^18,19^ These shocks could have created conditions for profit spikes independent of funding levels. Finally, the acquisition of Puerto Rico's 3 largest MA insurers by national corporations in 2021-2022 represents a market-structure shift that may have altered how revenues are allocated between medical care, administration, and profits.^20-22^ As of January 2026, these 3 companies—MMM, Triple-S, and MCS—are the only insurers remaining in the MA market for Puerto Rico.^23^

While health insurance performance has been understudied in Puerto Rico, reports from health economists^15^ and newspaper articles^24^ have highlighted the impact these corporations have had on the healthcare financing of the archipelago. Many patients, healthcare professionals, and academics argue that the healthcare system in Puerto Rico is in a state of crisis due to the control of private insurers in Medicaid and Medicare.^25-27^ At the same time, insurers contend that persistent federal funding disparities have undermined their ability to operate sustainably which causes the healthcare system to be more fragile.^28^ Therefore, this study aims to expand the knowledge of the MA program’s financial trends and evaluate the association of the funding disparity in the profitability of private insurers managing MA plans.

## Methods

### Study data

Data were obtained from Puerto Rico's Insurance Commissioner Office, which publishes annual financial statements for every health insurance company operating in the archipelago.^29^ These publicly available reports include each insurer's revenues, expenses, administrative costs, and net income (profits). They also detail the sources of revenue and delineate expenditures across key categories such as administrative costs, inpatient care, outpatient care, and prescription drugs. Public annual financial reports are mandatory for all insurance companies in Puerto Rico.

Data were manually extracted from each annual report into a consolidated dataset (2011-2022). Only the MA line of business for each insurer was included; commercial and Medicaid segments were excluded. The analysis focused on the 3 largest MA administrators (MMM, Triple-S, and MCS), which together covered approximately 92% of all Medicare beneficiaries in Puerto Rico in 2022. The extracted data were aggregated at the insurer-year level and cross-checked for internal consistency.

In addition, a MA funding disparity index was created to capture fluctuations in federal payment levels between Puerto Rico and the US states (including Washington, DC). This measure was derived using data from the Centers for Medicare & Medicaid Services Part C plan payment data, which provides per-beneficiary spending across all regions of the United States and territories.^30^ We combined the Part A/B payment and the rebate payment to estimate total MA payments for each jurisdiction and create the funding disparity index.

### Measures

We examined 4 primary measures for this study: MLR, ALR, profit margins, and the MA funding disparity index. Revenues (total income of insurers) were used to construct 3 financial ratios: MLR (medical expenditures divided by total revenues), ALR (administrative expenditures divided by total revenues), and profit margin (profits divided by total revenues). Each ratio was multiplied by 100 and expressed as a percentage. The MA funding disparity index represents the percentage by which average MA payments in Puerto Rico were below-average MA payments in the 50 states and Washington, DC. Higher values indicate a larger payment disparity, while lower values indicate that Puerto Rico's payments were closer to US payment levels.

### Analysis

First, we conducted summary statistics to describe the MA market in Puerto Rico. We included enrollment, total revenues, medical expenditures, administrative expenditures, profits, MLR, ALR, profit margin, and the MA funding disparity index. For each, we calculated the mean, minimum, and maximum across the 12 aggregate market years in order to characterize the scale, financial allocation patterns, and variability of the market before estimating the associations between the funding disparity and insurer financial outcomes.

Second, we used time-series graphs to examine longitudinal trends and co-movement between Medicare funding disparity and the financial measures. Using Microsoft Excel, visualizations for MLR, ALR, profit margins, and the MA funding disparity index fluctuations from 2011 to 2022 were created to evaluate their temporal alignment and present a time-series tracing profit margins alongside external shocks and market-structure changes affecting insurer profitability.

Third, we conducted separate ordinary regression analyses with the aggregate MA market-year dataset. We examined the association between the Medicare funding disparity and each financial outcome: MLR, ALR, and profit margins. We then estimated a second model using the 1-year-lagged MA funding disparity index to evaluate whether payment disparities were associated with financial outcomes in the following year. As a sensitivity analysis, we also estimated insurer-level models to assess whether associations between the MA funding disparity index and financial outcomes were similar to the aggregate market-level data.

All regression models used heteroskedasticity-robust standard errors to account for potential differences in the variability of financial outcomes across observations. Regression coefficients represent the percentage-point change in the financial outcome associated with a 1-percentage-point increase in the MA funding disparity index. Analyses were conducted using Stata 18.0 with a statistical significance threshold of *P* < .05.

### Limitations

This study has some limitations. First, the observational design of this study does not allow for causal inference. Although the regression analyses assess associations between MA funding disparities and insurer financial outcomes, changes in MLR, ALR, or profitability may also be impacted by other factors occurring during the study period. Second, our measures depend on insurer-reported financial statements filed with Puerto Rico's Insurance Commissioner Office. Although we cross-checked internal consistency, estimates of MLR, ALR, and profit margins could be biased by variation in reporting practices, accounting conventions, and potential reclassification of expenses. Third, the analysis does not separately account for beneficiaries' risk scores, which influence MA payments. However, this limitation primarily affects the estimated level of the MA funding disparity index rather than insurers' financial performance measurements. Finally, the study focuses on financial performance and does not directly measure downstream consequences for beneficiaries, such as access to health services, out-of-pocket costs, quality of care, or health outcomes. The findings should be interpreted as evidence about financial allocation patterns rather than health quality or outcomes effects.

## Study results

As shown in Table 1, mean enrollment across the 3 largest MA insurers was 485 400 members, ranging from 322 442 to 619 941, while annual member-months averaged 5.75 million and ranged from 3.77 million to 7.37 million. Mean annual revenues were $4.92 billion (range: $3.19–$7.67 billion), mean medical expenditures were $4.15 billion (range: $2.71–$6.72 billion), and mean administrative expenditures were $599.6 million (range: $364.9–$897.8 million). Annual profits averaged $83.8 million and ranged from a $23.6 million loss to $218.8 million in profits.

**Table 1:** Summary statistics for Puerto Rico's Medicare Advantage market, 2011-2022.

Measure	Mean	Minimum	Maximum
Enrollment, members	485 400	322 442	619 941
Member-months, millions	5.75	3.77	7.37
Total revenues, US$ millions	4915.9	3189.2	7671.0
Medical expenditures, US$ millions	4152.6	2708.6	6723.2
Administrative expenditures, US$ millions	599.6	364.9	897.8
Profits, US$ millions	83.8	−23.6	218.8
Medical loss ratio, %	84.6	80.1	87.6
Administrative loss ratio, %	12.3	10.9	13.6
Profit margin, %	1.7	−0.7	3.3
Funding disparity index, %	37.0	27.7 (closest to US MA payments)	42.6 (furthest from US MA payments)

Abbreviation: MA, Medicare Advantage.

The aggregate MLR averaged 84.6% (range: 80.1%-87.6%), the ALR averaged 12.3% (range: 10.9%-13.6%), and the profit margin averaged 1.7% (range: −0.7%-3.3%). The MA funding disparity averaged 37.0%, ranging from 27.7%, when Puerto Rico payments were closest to US payment levels, to 42.6%, when the disparity was greatest.

**Figure 1.**
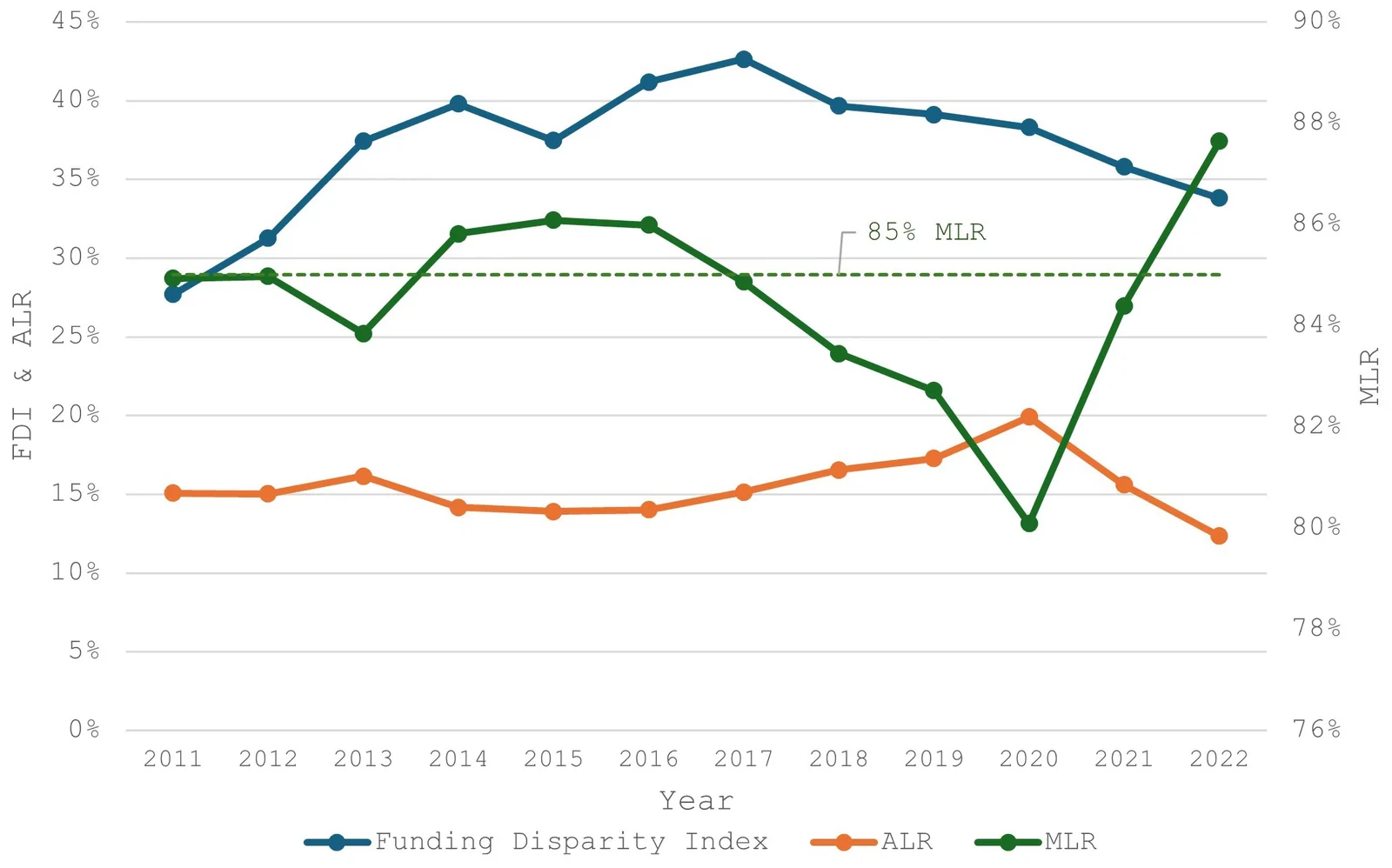
Administrative and medical loss ratios vs Medicare funding disparity in Puerto Rico's Medicare Advantage market, 2011-2022. Notes: FDI indicates funding disparity index; ALR, administrative loss ratio; and MLR, medical loss ratio. The horizontal dashed line marks the 85% MLR floor implemented in 2014 under the Affordable Care Act.

Figure 1 presents the annual medical and ALRs alongside the MA funding disparity index. The aggregate MLR was 84.9% in 2011 and remained near that level through 2013. It increased to 85.8% in 2014 and remained above 85% through 2016. The ratio then declined to 84.9% in 2017, 83.4% in 2018, and 82.7% in 2019 before reaching its lowest value, 80.1%, in 2020. It subsequently increased to 84.4% in 2021 and 87.6% in 2022. The ALR increased from 11.4% in 2011 to 13.5% in 2014. Then, it reached a study-period high of 13.6% in 2020. The ratio then declined to 11.2% in 2021 and 10.9% in 2022.

The MLR and ALR did not move together consistently with funding disparity throughout the study period, although several periods showed temporal alignment. Most notably, from 2017 to 2020, the funding disparity narrowed, while the MLR declined and the ALR generally increased, suggesting that improvements in relative MA payments during this period were not accompanied by a greater share of revenues being allocated to medical expenditures. After 2020, however, this pattern reversed: the funding disparity continued to narrow, while the MLR increased and the ALR declined. Overall, the visual trends suggest some correspondence between changes in the funding disparity and insurers' allocation of revenues, particularly administrative spending, but the relationship was not consistent across all years. A descriptive comparison of aggregate MA revenues and the funding disparity index is presented in Appendix A. Aggregate MA revenues increased consistently over the study period, while the funding disparity index fluctuated substantially, suggesting that changes in the funding disparity did not correspond with changes in insurer revenues.

**Figure 2.**
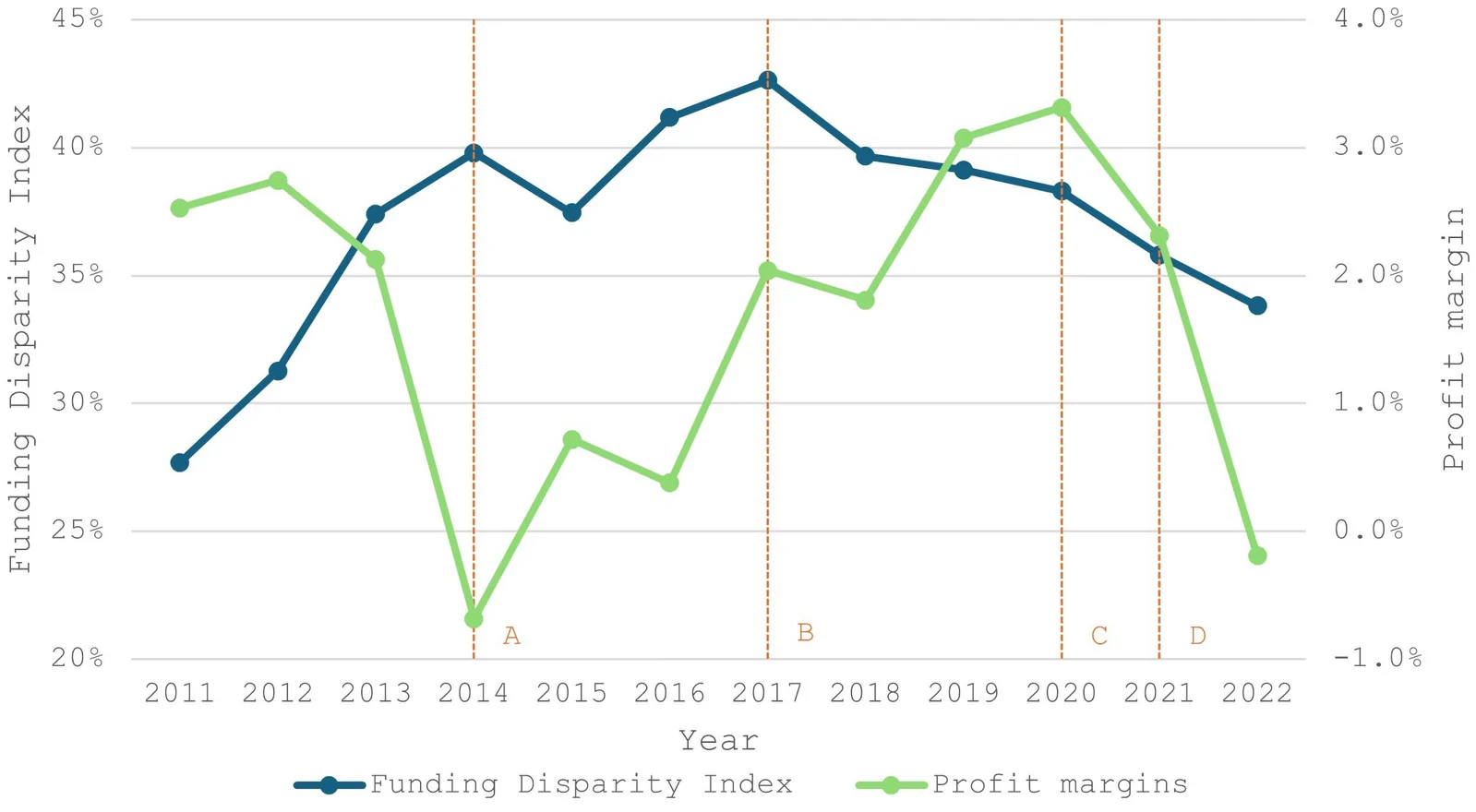
Medicare advantage profit margins, funding disparity, and selected external events in Puerto Rico, 2011-2022. Notes: The dashed vertical lines represent selected external events affecting Medicare Advantage: A, Affordable Care Act implementation; B, Hurricane María; C, COVID-19 pandemic; and D, acquisitions of Puerto Rico Medicare Advantage insurers by national firms.

Between 2011 and 2022, trends in the Medicare funding disparity did not consistently predict fluctuations in insurer profit margins in Puerto Rico. While this pattern appeared in certain years, it did not consistently hold across the entire period. Figure 2 shows that from 2016 to 2017, the funding disparity between Puerto Rico and the United States widened from 41.2% to 42.6%, reflecting lower MA payments to Puerto Rico's plans, yet insurer profit margins increased more than 5-folds from 0.38% to 2.04%, contradicting the assumption that underfunding directly reduces profitability. Similarly, between 2017 and 2018, the funding gap narrowed (from 42.6% to 39.7%), but profit margins decreased (from 2.04% to 1.81%), showing no immediate financial benefit from a narrower gap in funding between Puerto Rico and the US mainland. A similar divergence occurred between 2021 and 2022, the funding disparity narrowed from 35.8% to 33.8%, indicating that Puerto Rico's MA payments moved closer to US payment levels, while the aggregate profit margin fell from 2.31% to −0.19%. These 3 periods suggest that short-term changes in federal payment rates are not the sole determinant of insurer financial performance in Puerto Rico.

When comparing profit margins to external shocks, we found that profit margins were above 2% before the implementation of the ACA 85% minimum MLR requirement in 2014 (Line A) peaking at 2.75% in 2012. From 2014 to 2016, profits remained relatively low with negative margins in 2014 (−0.68%) and profit margins below 1% in 2015 and 2016. Following Hurricane María (Line B) in 2017, profit margins rose and peaked in 2020 at 3.32%, coinciding with the COVID-19 pandemic period (Line C). In the final year of the study, insurers reported a negative profit margin at −0.19% following the acquisition of major insurers in PR by national corporations (Line D), suggesting a possible change in their operational strategy.

Table 2 presents the MA market-aggregate models. We found that the funding disparity was not significantly associated with MLR (β = −.063; *P* = .655) or profit margin (β = −.075; *P* = .502). However, a wider funding disparity was associated with a higher ALR (β = .140; *P* = .041), suggesting that annual variation in the funding gap was more closely related to administrative spending than to medical spending or profitability.

**Table 2:** Associations between Medicare Advantage funding disparity index and financial outcomes in Puerto Rico, 2011-2022.

Financial outcome	Same year β	*P* value	1-year lag β	*P* value
MLR	−.063	.655	−.006	.910
ALR	.140	.041*	.054	.022*
Profit margin	−.075	.502	−.038	.328

β represents the percentage-point change in the financial outcome associated with a 1-percentage-point increase in the Medicare Advantage funding disparity. One-year lag β uses the funding disparity from the preceding year.

Abbreviations: ALR, administrative loss ratio; MLR, medical loss ratio.

^*^ *P* < .05 was considered statistically significant.

The 1-year-lagged models showed a similar pattern. The prior-year funding disparity was not significantly associated with MLR (β = −.006; *P* = .910) or profit margin (β = −.038; *P* = .328), but was positively associated with ALR (β = .054; *P* = .022). Overall, the aggregate results provide little evidence that changes in the funding disparity systematically affected insurer profitability, while indicating a more consistent relationship with administrative spending.

It is important to mention that insurer-level sensitivity analyses showed no significant association between ALR and funding disparity (Appendices B and C). However, among the financial outcomes examined, the ALR estimates had *P* values closest to the .05 significance threshold.

## Discussion

As MA serves as de facto Medicare payer for older adults in Puerto Rico, MA leaders point to relatively lower capitation rates as a driver of underinvestment in the territory's medical system.^28^ The issue of relative underfunding increasingly shapes proposals to reduce benefits and seek higher federal payment rates as a means to enable MA plans to spend more on medical services and to shore up insurer solvency. In this study, we examine plan financial reporting using insurer financial statements from 2011 to 2022 and a MA funding disparity index. We find that changes in the funding disparity aligned more consistently with shifts in ALRs than with insurer profit margins and MLR. By contrast, plan profitability was relatively volatile over the 12 years studied and appeared to be driven by major external shocks that impact medical utilization rather than funding disparity.

Shifts in Puerto Rico's funding disparity were mirrored more clearly in how plans allocated revenue in their administrative expenses. Aggregate MLR ranged from 80.1% to 87.6%, reaching its lowest level in 2020 before increasing to 87.6% in 2022, while ALR ranged from 10.9% to 13.6%. The regression analyses identified an association between greater funding disparities and higher administrative spending shares. This could suggest that health insurance companies may reoptimize internal administrative expenses in response to changing payment levels. However, the absence of a corresponding association in the insurer-level models (Appendices B and C) suggests caution in interpreting this relationship.

Profit margins for MA insurers appeared to respond to the ACA requirement of spending at least 85% of premium revenue on medical care, rather than on administrative costs or profits.^31^ This regulation coincided with lower profit margins in the years immediately following implementation of ACA regulations, indicated by higher MLRs and a dip in profit margins. However, profits rebounded by 2017 and continued to rise until 2020, mirroring a fall in medical loss over this period.

If the funding disparity represented a quantitatively meaningful shock to insurers' profitability, one might expect to observe higher profit margins when the MA funding disparity index narrowed and vice versa, but it did not occur. Figure 2 showed no relationship between the MA funding disparity index and insurer profit margins, suggesting that changes in the funding disparity did not correspond with changes in profitability. In addition to the descriptive patterns, the regression analyses did not identify a statistically significant association between the funding disparity and insurer profit margins. The absence of a consistent relationship between funding disparity and profitability suggests that private insurers' performance is less sensitive to federal payment adjustments than to utilization changes and internal business strategies. The surge in profitability during the COVID-19 years, when service use dropped sharply, illustrates a possible relationship between utilization and profitability.^32^

During the study period, 2 major shocks occurred in Puerto Rico, driving the government and the population to provide less attention to private movements and more attention to emergency management.^33^ These 2 events are Hurricane María, which occurred in 2017, and the COVID-19 pandemic, which began in 2020. In the years when a shock happened, health insurance companies' profits soared, while their investment in medical services decreased compared to the years prior to the event. In both events, utilization of medical services decreased due to infrastructure destructions and quarantine mandates.^19,34,35^ After Hurricane María, access to medical services was limited for months, in part due to unsafe or blocked roads (eg, fallen trees or light posts and flooded streets) reduced operations at healthcare facilities or closures due to power outages, flooding, and damaged infrastructure.^34,36^ In 2020, the pandemic led to reduced healthcare visits from fear of contagion yet an increase in severe cases at hospitals from infection. These events did not stop the flow of Medicare funds to MA plans, coinciding unusually high profitability. However, because this study did not measure utilization or estimate the causal effects of these events, these relationships should be interpreted as descriptive and not causal.

The lack of a consistent relationship between funding disparities and profitability also highlights the importance of insurer-specific behavior and organizational structure. Insurer-level analyses produced differing associations, suggesting that insurers may respond differently to changes in payment conditions (Appendices B and C). It is also important to highlight that vertical integration within the market may complicate the interpretation of insurer-level financial statements because expenditures recorded by an insurance entity may include payments to their own provider organizations. This is important because financial returns may be retained elsewhere within the same corporate structure and therefore may not be fully reflected in the insurer's reported financial outcomes.

### Policy implications

The findings suggest that Puerto Rico's MA market operates in a context-dependent environment where profitability and care spending are only partially explained by funding disparity in the Medicare program. Consistent underfunding relative to the US states and DC, combined with a lack of oversight and a lack of strong competitive pressures may collectively create the enabling conditions for MA plans in the territory to prioritize administrative spending and investor returns over clinical investments. The absence of a significant relationship between the funding disparity and insurer profitability does not demonstrate that Puerto Rico's MA payment levels are adequate; it indicates that annual variation in the payment gap alone does not explain changes in insurer financial performance. In some years, insurers achieved higher profit margins even as the funding disparity widened. These findings suggest that lower MA payment levels in Puerto Rico should not, by themselves, be assumed to result in poorer insurer financial performance or to justify reductions in benefits or healthcare services without closer examination of insurers' overall financial condition.

Policymakers should be aware that additional funds do not necessarily translate into a higher percentage of funds allocated to medical services. Policy decisions should not be based solely on the assumption that the funding disparity adversely affects insurers' financial performance, as our findings do not demonstrate a clear or consistent relationship between the 2. Although increasing Medicare funding in Puerto Rico may help ameliorate health system problems, progress would be maximized if coupled with clear guidelines on allocation of funds between medical services, administrative bureaucracy, and profits. While the ACA already restrains the MLR, policymakers could explore raising the minimum share for medical loss (eg, from 85% to 90%) over the additional payments. Correspondingly, policymakers could also explore more closely regulating which activities and subsidiaries' payments fall under “medical” loss, as contractual relationships grow increasingly complex, and many health insurance companies are vertically integrated with providers and pharmacy benefit managers. Finally, federal regulators could promote and strengthen the Traditional Medicare program, particularly by reducing out-of-pocket costs to beneficiaries, to increase competition in Medicare and to ensure a greater share of overall Medicare dollars in Puerto Rico is allocated to direct medical services and less administrative activities.

## Conclusion

To our knowledge, this is the first study to examine the relationship between MA funding disparities and insurers' financial performance in Puerto Rico. We found that there was not a clear relationship between health insurance profit margins and funding disparity between Puerto Rico and the US states and DC. Yearly fluctuations in funding disparities track more closely with shifts in ALRs than with profitability. Profit margins show abrupt swings that align with external utilization shocks such as Hurricane María, the COVID-19 pandemic, and health insurance companies' acquisitions. These findings suggest that increasing federal Medicare payments to Puerto Rico, while important for equity, should be coupled with specific financial allocations to improve care access and system stability. Future studies should examine how funding disparities and insurer behavior translate into health outcomes and care access for beneficiaries.

## Supplementary Material

qxag242_Supplementary_Data
