# Supplementary material for: Does Medicare Advantage funding disparity affect managed care insurers' profitability? Evidence from Puerto Rico
Source: Health Aff Sch. 2026 Sep 4;4(9):qxag242. doi: 10.1093/haschl/qxag242 (PMC13587251; doi:10.1093/haschl/qxag242)
Supplement: Supplementary file 1 [file qxag242_supplementary_data.zip › Appendix.docx]

Appendix A: Revenues and Funding Disparity in Puerto Rico Medicare Advantage Market, 2011–2022

Funding Disparity Index

Revenues ($US billions)

| Year | Funding Disparity Index |
| --- | --- |
| 2011 | 27.693% |
| 2012 | 31.2668% |
| 2013 | 37.4114% |
| 2014 | 39.795% |
| 2015 | 37.454% |
| 2016 | 41.1781% |
| 2017 | 42.6289% |
| 2018 | 39.667% |
| 2019 | 39.121% |
| 2020 | 38.2961% |
| 2021 | 35.8001% |
| 2022 | 33.8177% |

|  | Revenues |
| --- | --- |
| 2011 | 3.18918 |
| 2012 | 3.38404 |
| 2013 | 3.4267 |
| 2014 | 3.46628 |
| 2015 | 4.16459 |
| 2016 | 4.37373 |
| 2017 | 4.53278 |
| 2018 | 5.09631 |
| 2019 | 5.94135 |
| 2020 | 6.59892 |
| 2021 | 7.14621 |
| 2022 | 7.67105 |

Appendix B: Associations Between Medicare Advantage Funding Disparity Index and Financial Outcomes in Puerto Rico, Insurer-Level Models, 2011–2022

| **Financial Outcome** | **Same year β** | **p-value** | **1-year lag β** | **p-value** |
| --- | --- | --- | --- | --- |
| MLR | -0.121 | .401 | -0.138 | .370 |
| ALR | 0.122 | .085 | 0.110 | .196 |
| Profit margin | 0.007 | .964 | 0.027 | .852 |
| Notes: These insurer-level sensitivity analyses assess whether the aggregate findings are consistent when observations are retained at the insurer-year level. β represents the percentage-point change in the financial outcome associated with a one-percentage-point increase in the Medicare Advantage funding disparity. 1-year lag β uses the funding disparity from the preceding year. *P<.05 was considered statistically significant. MLR: medical loss ratio; ALR: administrative loss ratio. | | | | |

Appendix C: Associations Between Medicare Advantage Funding Disparity Index and Financial Outcomes in Puerto Rico, Firm Fixed-Effects Models, 2011–2022

| **Financial Outcome** | **Same year β** | **p-value** | **1-year lag β** | **p-value** |
| --- | --- | --- | --- | --- |
| MLR | -0.121 | .369 | -0.138 | .349 |
| ALR | 0.122 | .122 | 0.110 | .216 |
| Profit margin | 0.007 | .961 | 0.027 | .840 |
| Notes: These firm fixed-effects sensitivity analyses assess whether the findings persist after accounting for time-invariant differences across insurers. β represents the percentage-point change in the financial outcome associated with a one-percentage-point increase in the Medicare Advantage funding disparity. 1-year lag β uses the funding disparity from the preceding year. *P<.05 was considered statistically significant. MLR: medical loss ratio; ALR: administrative loss ratio. | | | | |
